# “Dicing and Splicing” Sphingosine Kinase and Relevance to Cancer

**DOI:** 10.3390/ijms18091891

**Published:** 2017-09-02

**Authors:** Nahal Haddadi, Yiguang Lin, Ann M. Simpson, Najah T. Nassif, Eileen M. McGowan

**Affiliations:** School of Life Sciences, Faculty of Science, University of Technology Sydney, 15 Broadway, Ultimo, Sydney, NSW 2007, Australia; Nahal.Haddadi@student.uts.edu.au (N.H.); Yiguang.Lin@uts.edu.au (Y.L.); Ann.Simpson@uts.edu.au (A.M.S.); Najah.Nassif@uts.edu.au (N.T.N.)

**Keywords:** sphingosine kinase (SphK), isozymes, variant isoforms, cancer therapy, sphingosine 1 phosphate (S1P)

## Abstract

Sphingosine kinase (SphK) is a lipid enzyme that maintains cellular lipid homeostasis. Two SphK isozymes, SphK1 and SphK2, are expressed from different chromosomes and several variant isoforms are expressed from each of the isozymes, allowing for the multi-faceted biological diversity of SphK activity. Historically, SphK1 is mainly associated with oncogenicity, however in reality, both SphK1 and SphK2 isozymes possess oncogenic properties and are recognized therapeutic targets. The absence of mutations of SphK in various cancer types has led to the theory that cancer cells develop a dependency on SphK signaling (hyper-SphK signaling) or “non-oncogenic addiction”. Here we discuss additional theories of SphK cellular mislocation and aberrant “dicing and splicing” as contributors to cancer cell biology and as key determinants of the success or failure of SphK/S1P (sphingosine 1 phosphate) based therapeutics.

## 1. Introduction

**Figure 1:**
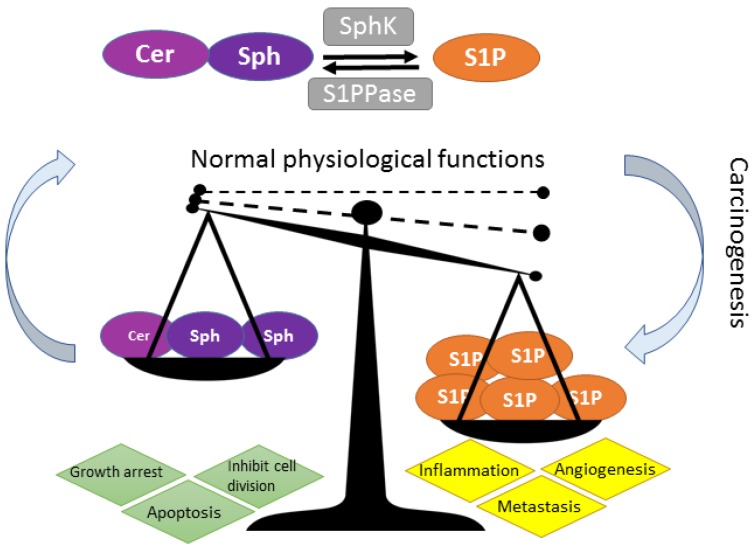
The SphK rheostat: tipping the sphingosine-sphingosine kinase (SphK)-sphingosine-1-phosphate (S1P) rheostat in favor of cancer. Ceramide (Cer) and sphingosine (Sph), upstream in the SphK rheostat, are pro-apoptotic while SphK conversion of sphingosine to S1P tips the balance in favor of cell survival and cell maintenance (as shown by the arrows and dashed lines). Imbalance (increase) in S1P expression, through overexpression of SphK activity, illustrated by dashed lines to solid line, is causally associated with cancer development, inflammation, angiogenesis and metastasis [1].

The theory that shifts in lipid metabolism drive oncogenesis and tumor recurrence, whereby cancer cells display alterations in fundamental cellular metabolism, is gaining credence [1,2,3,4]. Sphingosine kinase (SphK) is a lipid enzyme central to lipid metabolism, maintaining the cellular lipid homeostatic balance through its pivotal role in the conversion of sphingosine to sphingosine 1 phosphate (S1P) for maintenance of normal cellular function [5]. The SphKs are known to play pivotal roles in regulating many physiological pathways, and in the pathophysiology of various diseases, and the complexity of the SphK multi-functional signaling pathways are slowly being unraveled. Key cellular roles for SphK/S1P include the promotion of cell survival and proliferation, prevention of apoptosis, maintenance of vascularization and stimulation of angiogenesis for tissue regeneration in tissue damage, metabolic rewiring, and metabolic stability [5,6,7,8,9]. While these actions are important for cellular function, undesirable consequences of these cellular functions, when not restrained, underpin the mechanisms of oncogenesis, including uncontrolled cell division, pro-inflammatory responses, cell invasion and metastasis (Figure 1) [3,10].

There is a strong causal association between adverse, or overactive, SphK/S1P signaling and cancer, and there are extensive recent reviews on the importance of maintaining the balance of the SphK-S1P rheostat in the prevention and/or development of cancer [11,12,13,14,15,16,17,18].

### 1.1. Importance of Isoenzymes (Isozymes) and Variant Isoforms in the Future of Cancer Treatment

Multiple isozymes occur mainly through gene duplication throughout evolution and each isozyme can produce many alternatively spliced transcripts and variant protein isoforms. Alternative splicing of a single gene allows for selective inclusion and exclusion of specific exons within a gene and provides for greater proteomic multiplicity and physiological functionality [19]. Dicing and splicing of introns and exons to produce variant isoforms is a common process occurring in 95% of all multi-exon genes [19]. Advantages of alternative exon splicing in different tissues allows for variations in protein–protein interactions with consequent modulation of specific interacting networks in the cell to allow variations in function [20]. On the other hand, a switch in splicing preference, aberrant splicing of isoforms, or loss of isoforms, have been shown to correlate with the development and/or progression of malignancy [21,22,23]. Therefore, targeting alternative splicing pathways may be a potential avenue for therapeutic intervention and identification of aberrantly spliced variants, and/or novel protein isoforms, which may be useful as a diagnostic biomarker in cancer. The SphK family of proteins, which is part of a much larger superfamily of structurally related lipid signaling kinases [24], is derived from alternate splicing of two different isozymes which lead to the expression of many variant isoforms that act to direct many physiological functions in the cell. There is strong evidence associating SphK overexpression and the development and progression of many different cancer types (Table 1).

In the absence of any known cancer-associated mutations in the SphK isozymes or isoforms, the term “oncogenic addition”, where the cancer cell becomes reliant on SphK-S1P signaling for survival, has been proposed [86]. The relevance of aberrant dicing and splicing of SphK1 and SphK2 isozymes and the production of variant SphK isoforms in the development and progression of malignancy is very much underexplored. This review highlights and discusses our current knowledge of the SphK aberrant signaling that may contribute or drive SphK-coupled oncogenicity with particular interest directed towards the current understanding of SphK isozymes, alternatively spliced isoforms “dicing and splicing” and the potential contribution of these isoforms to cancer cell biology and their influence on SphK/S1P based cancer therapeutics.

## 2. SphK Isozymes and Isoforms

### 2.1 Clarification of SphK Nomenclature

Due to the disparity in SphK nomenclature in the literature, in this review SphK1 and SphK2 are referred to as isozymes (isoenzymes) and the variant SphK1 and SphK2 isoform identities are derived from GenBank and the literature and are summarized in Table 2.

### 2.2. SphK1 and SphK2 Isozymes Are Transcribed from Different Genes and Evolutionary Conserved

It is less than 20 years since the human SphK isozymes were sequenced [88,89,90], substantiating evolutionary conservation across a wide range of organisms from mammalian species and plants, to yeast [91]. The first two human SphK isozymes described were SphK1a (isoform 3) [88] and SphK2a (isoform 2) [89]. Each isozyme is located on a different chromosome, SphK1 is located on chromosome 17 (17q25.2) and SphK2 is located on chromosome 19 (19q13.2) [89]. There is high sequence homology between the two SphK isozymes, believed to have evolved from a much larger superfamily of sphingosine and diacylglycerol (DAG) lipid signaling kinases, and not just a product of a simple gene duplication event [24,89,91]. The 3D structure of SphK, as illustrated by the SphK1a structure determined by Wang and colleagues, is very distinct from other protein kinase and lipid kinase families [92].

There is 47% identity on the N-termini and 43% identity in the C-termini of the SphK isozymes, with 80% similarity in their enzymatic activity [93]. Both SphK isozymes contain five conserved domains (C1–C5). The N-termini contains the C1–C3 and the C-termini contains the C4 and C5 domain [89,94]. Within the C1 to C3 of both SphK isozymes resides a DAG kinase catalytic domain, conserved across the DAG and ceramide kinases [95]. Although the binding site for ATP has been found to be present within the conserved C2 domain, structural studies of SphK1 have shown that all the C1–C5 motifs contribute to the ADP binding [94]. The C4 domain appears to be unique to the SphK family, setting SphKs apart from other lipids and enabling their unique ability to catalyze the conversion of sphingosine to S1P—thus making SphKs the sole source of S1P [94,96]. Albeit this C4 sequence has the greatest diversity between the SphK1 and SphK2 isozymes, the domain responsible for sphingosine binding, suggesting this domain is responsible for preferred sphingosine substrate specificity [94,96].

The major differences in the alignment of the two-original human SphK sequences are that SphK2 possesses an additional 236 amino acids at the N-terminal, a proline-rich insertion in the middle of the SphK2 C-terminal sequence [89,91,97] and a pro-apoptotic BH3 binding domain [98]. Although the crystal structure of SphK2 has yet to be identified, the unique upstream N-terminal region and the proline-rich insert of the SphK2 allow for major differences in conformational motility and protein-protein interactions between the two isozymes.

The extra sequence at the N-terminal of SphK2 provides for binding of a greater number of substrates such as the immunomodulatory SphK2 drug FTY720, which is now used in the clinic as a therapy for multiple sclerosis, and has already provided a prime example [34,99].

**Figure 2:**
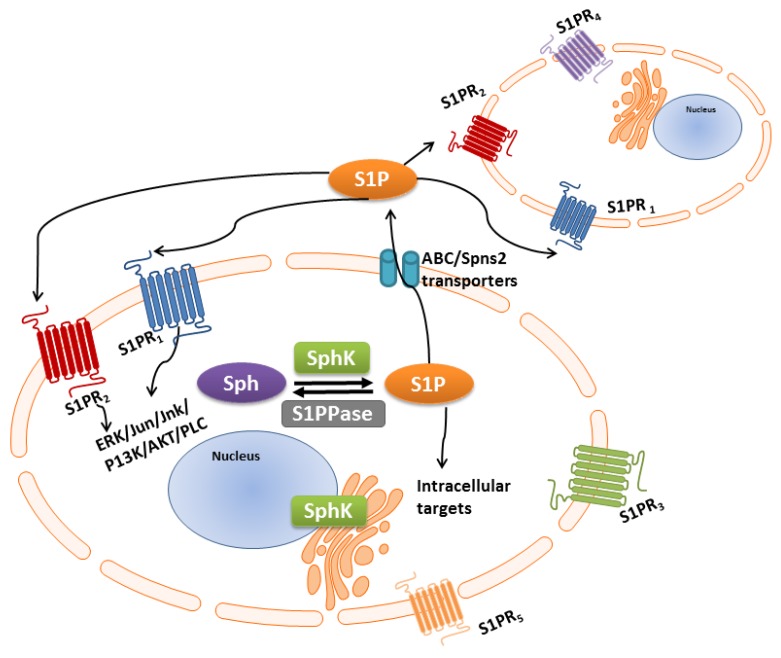
Complexity of SphK-S1P “inside-outside” signaling. SphK is a lipid enzyme catalyzing the phosphorylation sphingosine to its active form S1P. S1P can act in an autocrine or paracrine manner. S1P is exported extracellularly through the ABC [106] and Spns2 (Sphingolipid Transporter 2) transporter [107,108] transmembrane proteins or can act intracellularly on yet unknown targets. S1P extracellularly activity occurs through the binding of one or more S1P receptors (S1P1-5) located on the plasma membrane, which are coupled to different internal G proteins which in turn activate or inhibit downstream signaling pathways. This complex S1P signaling paradigm extends the repertoire of diverse cellular and biological processes of the SphK family of lipid isozymes and isoforms.

Given both isozymes catalyze the conversion of sphingosine to its S1P active form, it is intriguing to note that different and opposing functions have been assigned to the two SphK isozymes, SphK1 is pro-survival whereas SphK2 is pro-apoptotic [100]. Explanations for this dichotomy are partly found in the subcellular localization of these isozymes. SphK1 is predominantly cytoplasmic and upon agonist activation, such as growth factors [25], TNFα [101], and hormones [102], mainly mediated through ERK1/2 phosphorylation, which increases its activity and translocation to the plasma membrane [103]. Therefore, S1P can act as a dual “inside-outside” messenger. Intracellular S1P catalyzed by SphK can function as a second messenger inside the cell or S1P is exported outside the cell [9,34]. Secreted extracellular S1P binds directly to S1P receptors (S1PRs) on the cell surface and signals in an autocrine and/or paracrine manner, termed as an “inside-outside” mechanism of S1P action (Figure 2) [9]. Directed localization of SphK1 post stimulation by agonists has also been found to increase its catalytic activity [104], for example agonist-induced translocation to the nuclear/perinuclear space, potentially the endosomal compartments showed a major increase in SphK1 enzymatic activity [105].

On the other hand, SphK2 contains a nuclear localization sequence (NLS) in its unique 236 N-terminal allowing both nuclear and cytoplasmic sub-cellular localization depending on the cell milieu [97,109]. Inside the nucleus, S1P produced by SphK2 activation can bind and inhibit histone deacetylases (HDAC)1/2 [97,109,110] and human telomerase reverse transcriptase (tHERT) [111], directly modulating cell cycle signaling cascades. In the cytosol, SphK2-endoplasmic reticulum generated S1P appears to be directed into biosynthesis of pro-apoptotic ceramide [100]. Additional studies have also shown the extra SphK2 BH3-binding domain inhibits the anti-apoptotic protein BCL-x_L_ [97,98], thereby conferring pro-apoptotic or anti-tumorigenic functions to SphK2. It is noted that there is emerging evidence that a small proportion of SphK isozymes are released into the extracellular milieu and produce S1P directly in the extracellular environment [87]. The exact function of SphKs in the extra-cellular environment is still unclear.

Further insights into the structure-function of SphKs, mainly focusing on the SphK1 structure, can be found in the recent article by Adams et al. [112].

### 2.3. Lessons from the SphK “Isozyme” Knockout Mouse Models—From Mouse to Human

Most of the groundbreaking work in understanding the roles of mammalian SphK isozymes is from the SphKs knockout mouse models. Most importantly, although the two SphK isozymes have seemingly opposing functions, each have complementary and compensatory mechanisms. SphK1 and SphK2 knockout mice have provided exemplary models to study the effect on one or both isozymes in vivo [89]. During embryonic mouse development SphKs are temporally differentially expressed. SphK1 peaks at day 7 and decreases thereafter, whereas SphK2 gradually increases in expression up to day 17 [89]. Although SphK activity is present in all adult mouse tissues [113], a predominance of SphK1 is found in blood, lung, kidney, colon, spleen and lungs [113], whereas SphK2 predominance occurs in liver, kidney, brain, and heart [89]. In addition to differences in tissue distribution and sub-cellular localization, differences are observed in isozyme kinetic properties in response to alteration in ionic strength and detergents, and differences in their preferred sphingosine substrates, whereby SphK1 has a preference for d-*erythro*-sphingosine and d-*erythro*-dihydrosphingosine over other substrates, where SphK2 also phosphorylates phytosphingosine [104]. Albeit major differences are observed in SphK isozyme expression in both embryological and adult tissues, ablation of one of the isozymes is not embryonically lethal, mice are viable and fertile with no obvious pathophysiology [114,115,116,117]. These findings in SphK1^−/−^ and SphK2^−/−^ mouse models clearly demonstrate that the two isozymes have overlapping functions in development. In reality, both SphKs have been found in the extra-cellular environment, therefore able to generate S1P both intra- and extra-cellular thus expanding their repertoires and rapidity of signaling events [87]. In contrast, when both SphK isozymes are ablated mouse embryos do not survive, presenting with neurological and vascular developmental defects, thereby determining an undisputable role for SphKs/S1P signaling in mammalian development [118].

On further scrutiny, pathological differences in the SphK1^−/−^ and SphK2^−/−^ mice have started to emerge. A few examples include, mice deficient in SphK1 were rendered lymphopenic by FTY720 [115], endogenous SphK1 demonstrated a protective role in renal ischemia whereas SphK2 had a detrimental role [119], and in separate studies SphK1^−/−^ mice demonstrated a very poor survival following cardiac arrest [120]. Recently, mice lacking SphK2 were found to have 85–90% reduction of brain S1P and impairment of contextual fear memory [121]. A breakthrough finding presented SphK2/S1PR2 signaling, not SphK1, as an important enzymatic pathway involved in regulating hepatic lipid metabolism [122,123,124,125,126]. Dysregulation of the SphK2/S1P2 signaling pathway in mice fed on a high-fat diet were more susceptible to the development of fatty liver and related diseases through regulation of conjugated bile acids, important hormones produced during the feed/fast cycle [126]. Impaired bile formation and flow (cholestasis) has been associated with increased SphK2/S1P/S1PR2 activation and blocking this pathway with the specific S1PR2 inhibitor, JTE-013, reduced cholestatic liver injury through reduction of total bile acid levels in serum [124].

In the context of cancer development, there is some suggestion that SphK1 depletion may have some protective effect against the development of some cancers, for example SphK1^−/−^ mice were less inclined to develop colon cancer [67,127]. Treatment of tumor-bearing mice with SK1-I (SphK1 inhibitor), decreased S1P levels in both the circulation and in the tumor and significantly decreased the tumor size, angiogenesis and lymphangiogenesis [36]. In SphK2^−/−^ mice, SphK1 was upregulated to compensate for the loss of SphK2 associated with increased circulating S1P [127]. The increased S1P levels were associated with inflammation and risk of colitis-associated cancer [127].

The lessons we may take from these mouse studies are that although there are no overt physiological differences observed when one SphK isozyme is depleted, there are downstream consequences for how the body compensates for this loss and potentially increased vulnerability to cancer and other diseases.

### 2.4. SphK1 and SphK2 Isozymes Transcribe Multiple Variant Isoforms 

Sphingosine kinase isozymes have multiple alternatively spliced isoforms, which are listed in Table 2. The variant isoforms of both SphK1 and SphK2 identified provide greater functional diversity to this lipid kinase family. While many alternative human SphK variant isoforms have been identified their characterization and function is still in its infancy. Our knowledge of SphK isoform specificity and function in humans is severely limited, mainly due to the strong overlapping and compensatory roles of the isozymes and isoforms in normal physiology. All the SphK isozymes and isoforms catalyze the same reaction (sphingosine-S1P) making it extremely difficult to extrapolate individual and compensatory functions for each of the isozymes and more so for the isoforms. Most of the early studies on human SphK1 and SphK2 did not specify the isoform examined, and as the S1P activity has been shown to be similar between the different isoforms it has not been a high priority research area. Only a few studies have focused on the differences between the SphK isoforms and these have focused on the major SphK1a and SphK1b and the SphK2-S and SphK2-L. In addition, although the SphK2a gene was cloned in both human and murine cells in 2000 [89], the SphK2 larger isoform SphK2-L (SphK2b) was not found in mice [128]. Since SphK2 isoform 2 (b) appears to be specific to humans, studies on this isoform 2b have been performed in cell lines only [97]. Currently only the overexpression of each human SphK “isoform” in cell culture has shed some light into how they control different signaling pathways within the cell, and the functional consequences of overexpressing SphK individual isoforms against a background of endogenous SphK expression.

#### 2.4.1. SphK1 Variant Isoforms—Differences in Dicing, Splicing and Localization

**Figure 3:**
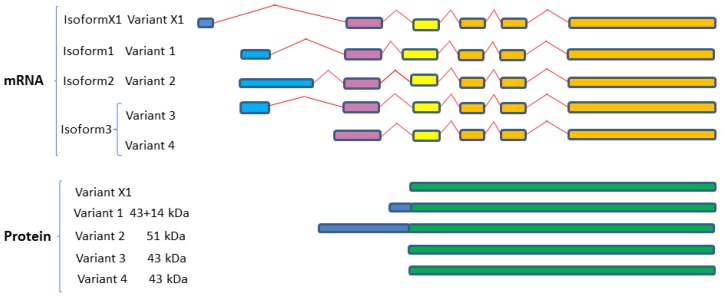
Variant splicing and dicing of human SphK1 isoforms. Schematic diagram of SphK1 splice variants and protein isoforms based on mRNA and protein sequences acquired from GenBank. Colored boxes are representative of different RNA fragments and protein fragments, same color boxes are originated from the same origin of DNA sequences and are identical/similar sequences. Expression from four variant mRNA transcripts (variants 1–4) results in three SphK1 isoforms (isoforms 1–3). A predicted fifth human SphK1 splice variant (variant X1), based on sequence prediction methods, results in a predicted fourth isoform (isoform X1) (annotated using gene prediction software and further evidenced by mRNA and EST). SphK1 sequences were aligned using Clustal Omega (V1.2.4) multiple sequence alignment (Available online: http://www.ebi.ac.uk/Tools/msa/clustalo/).

Three unique SphK1 isoforms have been characterized in humans which differ only in their N-terminal region (Table 2; Figure 3). Isoform 2 is the longest isoform with a molecular weight of 51 kDa, whereas the shortest isoform is isoform 3, 42.5 kDa, and isoform 1 has an extra 14 amino acids, 43.9 kDa. In addition, NCBI reports a predicted variant known as variant X1 which is not identified as protein and has been annotated using a gene prediction method and supported by mRNA and EST evidence. These isoforms are the result of alternative splicing variants, variant 1 and variant 2 encode isoforms 1 and 2 respectively, while variant 3 and variant 4 both encode isoform 3 (Figure 3). Distinguishing isoforms 1 and 3 on SDS PAGE is not plausible as they have a very similar molecular weight, while isoform 2 with an extra 86 amino acids is easy to be separated [129,130]. Comparison of the mammalian SphK1 and Sphk2 indicated that they all contain five highly conserved regions involved in the ATP binding and catalytic conversion of sphingosine to S1P.

Only a few studies have shown the localization of different isoforms in various tissue types. Most of the studies used the overexpression of the isoform 1 or 2 in human cells for characterization and determination of their biological significance [129,130,131,132]. Moreover, most of these in vitro studies used the SphK1 isoform 1 to study the function and structure of the SphK1 [111]. Both isoforms 1 and 2 translocate to the cell membrane, interestingly, the SphK1a (isoform 1) has been shown to be secreted from cells with more involvement in S1P/S1PR1 extracellular activity compared to SphKb (isoform 2) and SphKc (isoform 3) which appeared to remain mainly in the plasma membrane [132].

#### 2.4.2. SphK2 Variant Isoforms—Differences in Dicing, Splicing and Localization

**Figure 4:**
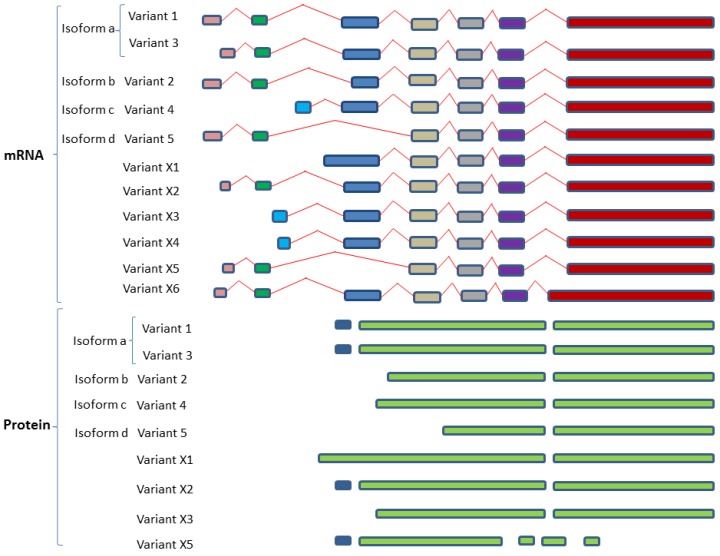
Variant splicing and dicing of human SphK2 isoforms. Schematic diagram of SphK2 variant isoforms based on mRNA and protein sequences acquired from GenBank. Colored boxes are representative of different RNA fragments or protein fragments, same color boxes are originated from the same origin of DNA sequences and are identical/similar sequences. The various described isoforms are derived from the expression of alternatively splicing SphK2 transcripts. Four protein isoforms have been identified for SphK2 and these express five SphK2 transcript variants. Human SphK2 isoform a (SphK2a/SphK-L) and SphK2 isoform b (SphK1b/SphK2-S) have been confirmed at the protein level. SphK2c and SphK2d protein translation has not been identified in humans to date. Variants X1-X5 were derived by automated computational analysis using gene prediction methods (Gnomon). Uniporter IDs are reported for variants SphK2 d, X5 and X6. All SphK2 sequences were aligned using Clustal Omega (V1.2.4) multiple sequence alignment (Available online: http://www.ebi.ac.uk/Tools/msa/clustalo/).

To date, five isoforms have been defined for SphK2 (Table 2; Figure 4): isoform 1 (a); isoform 2 (b); isoform 3 (a); isoform 4 (c); and isoform 5 (d). Variant 1 and 3 both represent the longest isoform identified as SphK2-a. Variant 2 encodes isoform SphK2-b which is shorter compared to variant 1. Both -a and -b isoforms have been confirmed at the protein level with 36 extra amino acids at N-terminal for -b. While the SphK2 -c and -d isoforms have been listed in GenBank to-date no translated protein has been identified [128] Similar to SphK1, SphK2 isoforms contain 5 highly conserved C-regions (C1–C5), however, isoform SphK2d does not contain the C4 and C5 regions. The C4 and C5 domains encompasses the sphingosine binding and catalytic properties, hence if this putative isoform was expressed as a protein, it would be catalytically inactive [128]. Furthermore, isoform 1a subcellular localization is in the cytoplasm and the membrane, while isoform 2b is located at the lysosome membrane.

### 2.5. SphK “Isoform” Specificity—Lessons from the Mouse Model

Our knowledge of SphK isoform specificity and function in humans is severely limited, mainly due to the strong overlapping and compensatory roles of the isozymes and isoforms in normal physiology. Most functional information of specificity of SphK1-isoform enzymatic traits comes from mouse SphK1 (mSphK1) models and cell lines. Lessons from the mouse reveal some promising differences in mSphK1a and mSphK1b isoform-specific molecular characteristics, including mobility on SDS-PAGE, stability, membrane localization and oligomerization and degradation properties [133]. Mainly, the differences in the N-terminal plays an important role in differences in specificity in enzymatic traits between the two mouse SphK1 isoforms, however, due to the vast difference in N-terminal sequence homology between mouse and humans we cannot easily translate these findings to human SphK isoform differences. As mentioned earlier, both SphK2 isoforms are not present in mice; therefore, comparative SphK2 isoform functional studies in vivo are not available. A comprehensive update on the discovery and comparison of the human and mouse SphK isoforms has recently been published [12].

## 3. S1P-S1PR1-5 Signaling Rheostat—Multiple Functions, Multiple Signaling 

SphKs are the only kinases that, in an ATP-dependent manner, phosphorylate sphingosine to its pro-active form S1P, initiating a cascade of signaling events inside and outside of the cell promoting cell survival and proliferation [134]. This action is reversible by dephosphorylation of S1P by S1P phosphatases or irreversibly by phosphate-dependent S1P lysate [134]. Not only do SphKs have multiple isozymes and with many variant isoforms, this dual “inside-outside” action of S1P allows for the diverse signaling events associated with cellular maintenance and function. It is unsurprising aberrant SphK/S1P/S1PR signaling has gained much credence in cancer targeting.

### S1P/S1P Receptor “Inside-Outside” Signaling 

As briefly mentioned, extracellular S1P modulates different signaling cascades through binding to one of the five S1P receptors (S1PR1-5) (Figure 2) on the plasma membranes, otherwise known as S1P-specific G-protein coupled transmembrane receptors (GCPRs), previously known as endothelial differentiation gene (EDG) receptors [135,136]. Not all S1PRs are expressed on the plasma membrane on the same cells at the same time, and during different stages of maturation, thereby permitting different cellular responses and different tissue functions in response to S1P activation [137]. S1PRs 1-2-3 are also widely distributed on most tissue types whereas SIPR4 and S1PR5 are more selective in tissue distribution. S1P receptors have overlapping signaling cascades with the potential for compensatory cellular functions [138].

As stated, S1PR1 is ubiquitously expressed on most tissue types, again defining a critical functional role for this receptor [139]. S1PR1 is essential for survival as mouse embryos with S1PR1 knockdown do not survive due to immature blood vasculature formation leading to intrauterine death [140]. Impairment of S1P1, exacerbates autoimmune inflammation in the brain [141] and leads to leaky vasculature [142]. In contrast, ablation of individual S1PR2 and S1PR3, are not embryonically lethal and do not demonstrate any overt abnormal phenotype at birth [143]. Co-expression of S1PR1, S1PR2 and S1PR3 increase vascular integrity, reviewed in [137]. However, double-null S1PR2 and S1PR3 embryos resulted in impairment of vascularity and partial embryonic lethality, suggesting overlapping functions of S1PR2 and S1PR3 [143]. The S1P4-5 receptors have more selective tissue distribution compared to the more prominent S1P1-3 receptors, although S1P 4 and 5 receptor deletions are not lethal [140]. S1PR4 is primarily located on peripheral leukocytes and lymphoid tissues [144], and has the potential to transduce a chemotactic response through inhibition of cell proliferation and effector cytokines and enhancement of suppressor cytokines [145]. Interestingly, S1PR5, mainly localized in the nervous tissue [146], has also been found on spleen [147] and natural killer cells [148], and has recently been associated with mitotic progression [149]. S1PRs are also located inside the cell, although not much information is available in intra-subcellular localization and function. Inside the cell S1P can be manufactured and located in different sub-cellular compartments, again allowing for greater functional diversity, dependent and independently of S1PRs [137]. Pyne and colleagues demonstrated S1PR5 localization in the centrosomes in the nucleus associated with cell division [150], S1PR2 localized to the nucleus preventing cell proliferation [151], and S1P catalyzed by SphK2 bound to S1PR4 to prevent trans-sub-nuclear cellular location of S1PR2 and promoting cell proliferation in estrogen receptor negative MDA-MB-231 breast cancer cells [151]. It is noted that not all physiological functions of SphK-S1P are dependent on S1P receptor binding [152]. S1P has been shown to directly interact with tumor necrosis factor receptor associated 2 (TRAF2), thus acting as a localized second messenger within the cell [8]. As briefly mentioned, S1P produced by SphK2, binds to and modulates HDAC1/2 and hTERT function in the nucleus, and in the mitochondria, S1P, produced by SphK2, binds to prohibitin 2 and assists in the regulation of the respiratory complex IV assembly [153].

The multifactorial complexity of the SphKs/S1P/S1PRs signaling in normal physiology and cancer is slowly being unraveled. Each step, and each signaling molecule, in the SphKs/S1P/S1PR signaling cascade is currently being scrutinized as a potential target for cancer therapy (Table 3, [12]). Emerging evidence on the studies of SphK isozymes in mice is that under normal physiological conditions there appears to be no obvious phenotypic differences in function when one of the isozymes (SphK1 or SphK2) is deleted, but in abnormal physiology, dysfunction of the isozymes, by deletion, has the potential to influence disease progression. The following section presents an updated overview on how SphK1 and 2 isozymes and isoforms are linked to carcinogenesis, development of resistance to cancer treatment and new cancer therapeutic targets.

## 4. Over-Active SPHK-S1P Signaling and Relevance to Cancer

Although there is a strong, indisputable, causal association between adverse SphK/S1P signaling and cancer, to date there have been no reports of SphK/S1PR mutations linked to cancer development and it has been suggested that cancer cells develop a dependence on SphK cellular signaling, referred to as “non-oncogene addiction” [86]. In addition, overactive SphK signaling is causally associated with treatment resistance, in particular endocrine resistant tumors [195]. In a recent review S1P levels was a good indicator of clinical outcome in breast cancer patients, increased S1P was associated with drug resistance to common drug therapies (hormone, herceptin (HER2) and chemo-therapies) [196]. To this end, novel inhibitors of SphK activity have been designed and successfully tested and characterized in human cancer cells and primary cancer cells from patient samples as listed in Table 3.

### 4.1. SphK1 Isozyme Is Overexpressed in Multiple Cancer Types 

Functionally SphK1 is the major isoform linked with many of the hallmarks of cancer and it has historically been identified as a major driver of cancer with a shorter overall survival in many human cancer patients [197], contributing to chemo-resistance and poor survival. Overexpression of SphK1 is linked to oncogenicity through various mechanisms such as imbalance of SphK1/S1P, enhancing oncogene Ras, promoting cancer stem cell proliferation to increase tumorigenesis, and imbalance between intracellular and extracellular S1P [15]. Aberrant SphK1 is also involved in the neovascularization of tumors involving paracrine angiogenesis and lymphangiogenesis [160,197,198]. Knockdown of SphK1 in breast and glioma cancer cells have been found to reduce migration and tube formation [197]. There have been numerous compounds designed to target SphK, albeit with limited efficacy in clinical trials [17]. However, given the importance of SphK1 in malignancy, it is anticipated that new SphK1 targets will be discovered, especially for hard to treat cancers that overexpress SphK1. For example, recently, Zhu et al. [75] found that a calcium and integrin binding protein CIB2 proved to be a novel and valuable target, which downregulated SphK1 signaling in ovarian cancer, suggesting CIB2 as a new therapeutic SphK1-targeted candidate for ovarian cancer.

### 4.2. SphK2 Isozyme—A Promising Cancer Therapeutic Target

Our knowledge of SphK2 isozyme and isoform diversity is less well studied as an oncogene compared to its counterpart SphK1. In the main, this is due to the overwhelming evidence in cancer clinical patient analyzes showing overexpression of SphK1 (Table 1) and the initial findings by Xia and colleagues that provided the evidence of SphK1 being oncogenic [101]. In contrast, historically, SphK2 has mainly been associated with apoptosis and cell death [100]. The line in the sand between the opposing functions of the two isozymes is unclear as both isozymes are enzymatic catalytic convertors of sphingosine to S1P, the bioactive lipid mediator of cell survival. In recent years, strong evidence supports the overexpression of SphK2 in many human cancers [199].

Observations by Pitson and colleagues [199] provide some evidence suggesting that levels of SphK2 dictate its pro-apoptotic and pro-oncogenic outcomes. When low, increased SphK2 (approximately 2.5-fold above normal adjacent tissue levels) is sustained, there is the potential for neoplastic transformation in these cells confirming a direct role in promoting oncogenesis, whereas in comparison, high levels of SphK2 may promote pro-apoptotic signaling [199]. These results add further complexity to the roles of SphK2 in cancer.

As recently reviewed by McGowan and colleagues, SphK2 is gaining acceptance as a valid cancer therapeutic target and specific SphK2 modulators (agonists and antagonists) appear to have increased efficacy in clinical trials for cancer treatment [12]. Specifically, the results from a recent phase 1 cancer clinical trial for the SphK2 inhibitor ABC294640 strongly supported the use of SphK2 inhibitor as an anticancer treatment [200], positively demonstrating ABC294640 pharmacologic inhibition of SK2 resulted in clear anticancer activity [200].

Indeed, the dichotomy of the individual roles of SphK1 and SphK2 are even more blurred when it comes to more aggressive cancers or metastasis and treatments which target SphK1 and/or SphK2. A few recent studies demonstrate the dependence on both SphKs/S1P activity for cancer survival and metastasis [37,81]. A recent study by Maiti et al. [37] demonstrated that a selective inhibitor of SphK2 (K-145) markedly attenuated epidermal growth factor (EGF)-mediated cell growth and survival of LM2-4 breast cancer cells, a cell line representative of the triple negative (progesterone/estrogen/herceptin 2 negative) breast cancers.

### 4.3. Targeting S1PRs in Cancer Therapy

There is no doubt that aberrant S1PRs expression plays an active role in cancer, making S1PRs modulators increasingly popular as cancer targets, we refer the reader to reference ([12] listed in Table 3, Figure 3). Not all S1PRs are equal in cancer development, as there is some controversy on the individual roles in S1PRs signaling in tumorigenesis. The mechanisms of S1PRs signaling and cancer have been extensively reviewed in [8,138]. Importantly, the S1P1 receptor is the most extensively studied S1P receptor due to its pro-tumorigenic properties, which include the promotion of tumor cell migration, invasion and the neovascularization required for tumor nutrition [201,202,203]. As such, a number of S1PR1-specific modulators (agonists and antagonists) and modulators that target multiple S1PRs that include S1PR1, have been designed that have potential as cancer therapies [8], however only a couple have made it into cancer clinical trials, reviewed in [8,12]. The essential nature of S1PR1 in normal cell physiology also adds to the difficulty of targeting this specific S1PR as a cancer therapy. S1PR1 modulators (Gilenya and ozamipod) are currently used in the clinic to treat autoimmune diseases, however some of the very unpleasant side effects of these drugs include transient bradycardia, macular oedema, and susceptibility to viral infections [18]. Less is known about the pathological role(s) of S1PR2-5, and therefore anti-S1P2-5 receptor modulators have not been as well explored in anti-cancer therapy. Controversially, in some studies S1PR2 has been shown to be anti-tumorigenic in both mice and cell culture studies [204,205,206,207], and in other studies pro-tumorigenic, and these findings are believed to be cell context specific [138]. High S1PR3 and S1PR4-5 are causally associated with poorer survival in breast cancer [138]. High S1PR5 expression has been associated with large granular lymphocytic leukemia [147].

Due to the discreet fundamental biological processes assigned to each S1PR, which have been described as having pro- or anti- apoptotic signaling depending on the cell type and cancer involved it has been highlighted that optimization of cancer treatment using anti-S1PR drugs would be necessary for each cancer type [208]. As noted in the review by Watters and colleagues [208], targeting S1PR1 in glioblastoma to decrease proliferation would have opposing results in T-lymphoblastic lymphoma. To this end, many new S1PR specific compounds are currently being tested and patented [209]. Thus, new cancer therapies have been designed to target the SphK isozymes and intermediaries in its downstream signaling pathway ([12], Table 3).

To-date SphK1 and SphK2 isozymes and the S1PRs have been the major focus of new designer therapies [17] due to the increasing evidence that over-active SphK-S1P signaling is an important driver for many human cancers, as tabulated in Table 1. The ongoing question, “should therapy be targeting SphKs or S1PRs”?

## 5. “Dicing and Splicing” Sphingosine Kinase Variant Isoforms and Relevance to Cancer

Due to the heterogeneity of cancers, the major problem with cancer treatments and the major cause of cancer-related deaths is resistance and recurrence to current therapies resulting in metastasis. Coming into the limelight as switches in cancer progression are the “dicers and splicers” of introns and exons, whereby aberrant splicing and the loss of expression of particular isoforms of importance are associated with malignancy [21,22,23]. SphK is no exception in this case.

### 5.1. Homing into SphK1 Isoform Expression in Anti-Cancer Targets

Although little is known about alternative splicing of SphK in cancer, we know that alterations in SphK isoform expression lead to changes the direction of SphK signaling pathways [35]. The additional N-terminal 86 amino acids of SphK1b allows for specific partner binding to this unique region. As described by Yagoub and colleagues [35], in general, isoform-specific interactions were more frequently observed with the SphK1b (51 kDa) isoform. This N-terminal amino acid extension of SphK1b also allows for conformational differences between the two major isoforms, thus also facilitating SphK1a, as well as SphK1b, isoform-specific partner interaction [35]. These results present a case for alterations in isoform abundance ratios conveying differences and similarities in SphK downstream signaling events. That said, we and others have shown that both isoforms have the same S1P activity and do not exhibit any overt phenotypic changes in cell morphology or function [35,129,130]. On further scrutiny, what we are finding is that changes in SphK1a and SphK1b expression levels can make cancer cells more vulnerable to treatment resistance. For example, in the immunoprecipitation study of Yagoub and colleagues, SphK1b interacted preferentially with dipeptidyl peptidase 2 (DPP2), a protein targeted in diabetic therapy and involved in the regulation of glucose metabolism [35]. Treatment with a DDP2/4 inhibitor in hormone-dependent breast cancer cells enhanced SphK1b expression with no change in SphK1a. Similarly, Pyne and colleagues demonstrated differences in the treatment response in prostate cancer depending on enhancement of specific SphK1 isoform expression [129,130]. Enhancement of SphK1b in androgen-independent prostate cancer cells altered anti-SphK drug efficacy [47,129,130]. Although there are no current studies exploring the expression of SphK specific isoforms in cancer patients, these initial in vitro studies in cancer cell lines, suggest that differences in SphK1 isoform expression may be relevant in anti-SphK/S1P/S1PR cancer based therapies.

### 5.2. SphK2 Isoforms as Anti-Cancer Targets

SphK2 isoform functions are even less characterized than the SphK1 isoforms. In human cancer cells, the longer SphK-L isoform appears to be the predominant isoform expressed [97]. In these studies, the two isoforms (SphK2-S and SphK2-L) where found in different subcellular locations, suggesting distinct functions [97]. There is a strong drive for SphK2 inhibitors given that they appear to be more efficacious than SphK1 inhibitors in in vitro cancer cell line studies and cancer clinical trial studies, in particular the SphK2 inhibitor ABC294640 (Table 4). Interestingly, from kinetic studies of the sphingosine analog, FTY720, it is the unique N-terminal extension of the longer SphK2 isoform (SphK2-L) which confers more efficient binding, resulting in increased efficacy compared to the shorter, SphK2-S, isoform [99]. Thus, a consideration of SphK isoform specificity may, in future, provide more precision therapeutic targets in the design of new SphK targeted cancer therapies.

## 6. Conclusions

Without a doubt, over-activity of SphK-S1P has a fundamental role in cancer progression. Overexpression of SphK1 is now becoming recognized as a diagnostic marker for many cancers, however, it may only have limited value as a prognostic indicator of treatment outcome. Despite successful anti-SphK1 inhibitor studies in vitro (Table 3), these compounds have not yet been translated into drugs for cancer treatment. Interestingly, anti-SphK1 agonists and antagonists are proving less efficacious than anti-SphK2 drugs. From our limited knowledge differences in the expression of individual SphK isoforms in human cancers may have consequences leading to either increased or decreased vulnerability to resistance to cancer treatments. To date, we have no information on the expression of the various SphK isoforms in normal and malignant human tissues, however what is emerging is that different tissues express different SphK isozymes and S1P receptors, and it is, therefore, not unreasonable to expect differential isoform expression through alternative splicing events dictated by the cellular and metabolic milieu. The possibility and consequences of SphK isoform instability in human cancers is yet unexplored, even though aberrant SphK isozymes and altered expression and sub-cellular location of isoforms was observed and, together, may contribute to cellular transformation and cancer. With current knowledge exposing the increasing complexity of SphK/S1P/S1PR signaling and the dependency on this signaling pathway in different cancer cell types, it is apparent that further study is of importance to characterize the specificity of SphK isoforms in cancer tissues when seeking new diagnostic targets and therapeutic interventions.

## Figures and Tables

**Table 1:** Overexpression of SphK is causally linked to cancer.

Cancer Sub-Type	Reference(s)
Breast	[25,26,27,28,29,30,31,32,33,34,35,36,37,38,39,40,41]
Prostate	[42,43,44,45,46,47,48,49,50,51]
Leukaemia	[52,53,54,55,56]
Lung	[57,58,59,60,61]
Pancreas	[62,63,64,65]
Renal	[66]
Colon	[67,68,69]
Ovarian	[70,71,72,73,74,75] *
Brain	[76,77,78,79]
Uterine Cervical	[80] *
Liver	[81,82,83,84,85] *

* SphK has been identified as potential diagnostic markers in human cancer patients; (Table updated from [12]).

**Table 2:** Nomenclature of SphK1 and SphK2 isozymes and protein isoforms.

Isozymes	Isoform Name	Isoform No.	Variant No.	GenBank Accession	Uniprot ID
Sphingosine kinase 1 (SphK1; SK1)	SphK1a	Isoform 3	Variant 3	NM_001142601	Q9NYA1-1
			Variant 4	NM_001142602	
			Variant X1 *	XM-005257766	
	SphK1b	Isoform 2	Variant 2	NM_182965	Q9NYA1-2
	SphK1c	Isoform 1	Variant 1	NM_021972	Q9NYA1-3
	SphK1a+14				
Sphingosine kinase 2 (SphK2; SK2)	SphK2a	Isoform 1 and 3	Variant 1	NM_020126	Q9NRA0-1
	SphK2-L		Variant 3 **	NM_001204159	Q9NRA0-3
	SphK2b	Isoform 2	Variant 2	NM_001204158	Q9NRA0-2
	SphK-S				
	SphK2c	Isoform 4	Variant 4	NM_001204160	Q9NRA0-4
	SphK2d	Isoform 5	Variant 5	NM_001243876	Q9NRA0-5
			Variant X1	XM_017027008	Q9NRA0-5
			Variant X2	XM_011527133	Q9NRA0-1
			Variant X3	XM_006723292	Q9NRA0-2
			Variant X4	XM_011527134	Q9NRA0-2
			Variant X5	XM_017027009	
			Variant X6	XM_017027010	

* Variant X1 has been annotated using gene prediction methods supported by mRNA and expressed sequence tag (EST) evidence. ** Variant 3 also has the Uniprot ID Q9NRA0-1. Note: The nomenclature in this Table is derived from the GenBank and Uniprot entries. In some studies, SphK1b is referred to as SphK1c [87], however for direct comparison between studies, the Genbank accession numbers are consistent between studies.

**Table 3:** SphK inhibitors tested in human cancer cells and primary human cancer cells.

SPHK Inhibitor	Cancer Cell Type	SphK Selectivity	References
B5354-c	Prostate (LNCaP, Du145 and PC-3)	SphK1	[45,154]
	Breast (MDA-MB-231)		
F-12509a	Leukaemia (HL-60, LAMA-84 and HL-60 MDR)	SphK1	[155]
	Chronic Myeloid Leukaemia blasts		
SK1-I	Glioma (U87MG, LN229 and U373) and Primary Glioma Cells (GBM6)	SphK1	[36,129,156,157,158,159]
	Leukaemia (U937, HL-60 and Jurkat) and Acute Myeloid Leukaemia blasts		
	Breast cancer (MDA-MB-231, MCF-7 and MCF-7 HER2)		
	Prostate (LNCaP)		
SKI-II	Prostate (LNCaP, C4-2B and PC-3)	SphK1 and SphK2	[43,160,161,162,163]
	Pancreas (Panc-1 and BXPC-3)		
	Bladder (T24)		
	Breast (MCF7)		
ABC294640	Pancreatic (clinical trial) and (Panc-1)	SphK2	[163,164,165]
	Colorectal (HT-29 and Caco-2)		
	Breast (MCF-7, MDA-MB-231)		
	Ovarian (SK-OV-3)		
	Prostate (DU145)		
	Kidney (A-498)		
	Melanoma (1025LU)		
	Bladder (T24)		
	Liver (Hep-G2)		
Safingol	Solid tumors (clinical trial)	SphK1 and SphK2	[8,165,166]
	Glioblastomas		
	Colorectal tumor		
	Colorectal (HCT116)		
	Adrenal cortical carcinoma		
	Sarcoma		
*N*,*N*-dimethyl-d-*erythro*-sphingosine (DMS)	Lung (A549)	SphK1 and SphK2	-
	Acute myeloid leukemia (HL-60, U937, CMK7)		[167,168,169,170,171,172,173]
	Chronic myeloid leukemia (JFP1, K562)		[174]
	Gastric (MKN45, MKN74, Kato III)		[175,176]
	Acute lymphoid leukemia (Jurkat)		[177,178]
	Lung (LU65, NCI-1169)		[175]
	Cervix carcinoma (KB-3-1)		[179]
	Colon (Colo205, SW48, SW403, SW1116, SW1417, HT29, LS174T, LS180, HRT18)		[169,175]
	Pheochromocytoma (PC-12)		[180,181]
	Prostate adenocarcinoma (LNCaP)		[182]
	Melanoma (M1733, F10, F1, BL6)		[175]
	Hepatoma (Hep3B)		[183]
	Epidermoid carcinoma (A431)		[169,175]
	Breast adenocarcinoma (MCF7)		[184]
l-*threo*-dihydrosphingosine (DHS)	Acute myeloid leukemia (HL-60, P388, U937, NB4) Lymphoma (WEHI-231)	SphK1 and SphK2	[171,172,185,186]
	Breast adenocarcinoma (MCF7)		[187,188]
	Hepatoma (Hep3B)		[183]
	Neuroblastoma (SH-SY5Y)		
	Melanoma (A2058, 939, C8161)		[188]
FTY720 (fingolimod)	Prostate (PC-3, LNCaP-C4-2B, and DU145)	SphK1	[189,190,191,192,193]
	Ovarian cancer (OV2008, IGROV-1, A2780, SKOV-3, R182)		
	Bladder (T24, UMUC3 and HT1197)		
	Glioblastoma (U251MG and U87MG)		
	Hepatoma (HepG2, Huh-7 and Hep3B)		
K145	Leukemia (U937)	SphK2	[194]

**Table 4:** SphK/S1P/S1PR drugs in clinical trials related to cancer treatment (Adapted from [12]).

Drug	SphK Selectivity	Indications	ClinicalTrials.gov Identifier	Phase
Safingol	Sphingosine derivative, PKC inhibitor	Solid tumors, combined with fenretinide Solid tumors, combined with cisplatin	NCT01553071 NCT00084812	I (Recruiting) I (Completed)
ABC294640	SPHK2 inhibitor	Pancreatic cancer	NCT01488513	I (Completed)
		Diffuse Large B Cell-	NCT02229981	I (Recruiting)
		Lymphoma		
		Kaposi Sarcoma	NCT02229981	II (Recruiting)
		Multiple Myeloma	NCT02757326	Ib/II (Recruiting)
		Carcinoma, Hepatocellular	NCT02939807	II (Recruiting)
Sonepcizumab (ASONEP)	S1P-specific monoclonal antibody	Advanced Solid tumors Renal Cell Carcinoma	NCT00661414 NCT01762033	I (Completed) II (Terminated)
Fingolimod	S1PR antagonist	Glioblastoma	NCT02490930	I (Recruiting)
